# Post-endotoxin exposure-induced lung inflammation and resolution consequences beneficially impacted by lung-delivered IL-10 therapy

**DOI:** 10.1038/s41598-022-22346-2

**Published:** 2022-10-15

**Authors:** Jill A. Poole, Rohit Gaurav, Aaron Schwab, Amy J. Nelson, Angela Gleason, Debra J. Romberger, Todd A. Wyatt

**Affiliations:** 1 Department of Internal Medicine, College of Medicine, University of Nebraska Medical Center, Omaha, NE USA; 2 Veterans Affairs Nebraska-Western Iowa Health Care System, Research Service, Omaha, NE USA; 3 Department of Environmental, Agricultural and Occupational Health﻿, College of Public Health, University of Nebraska Medical Center, Omaha, NE USA

**Keywords:** Cytokines, Inflammation, Immunology, Asthma

## Abstract

Although lung diseases typically result from long-term exposures, even a robust, one-time exposure can result in long-lasting consequences. Endotoxin is a ubiquitous environmental/occupational inflammatory agent often used to model airway inflammation. Using a murine model, the return to lung homeostasis following high dose inhalant lipopolysaccharide (LPS, 10–100 μg) exposure were delineated over 2 weeks. LPS-induced rapid weight loss, release of proinflammatory mediators, and inflammatory cell influx with prolonged persistence of activated macrophages CD11c^+^CD11b^+^ and recruited/transitioning CD11c^int^CD11b^+^ monocyte-macrophages out to 2 weeks. Next, lung-delivered recombinant (r) interleukin (IL)-10 was intratracheally administered for 3 doses initiated 5 h following LPS (10 μg) exposure for 2 days. IL-10 therapy reduced LPS-induced weight loss and increased blood glucose levels. Whereas there was no difference in LPS-induced bronchoalveolar lavage airway fluid cellular influx, total lung cell infiltrates were reduced (37%) with rIL-10 treatment. Post-LPS exposure treatment with rIL-10 strikingly reduced lavage fluid and lung homogenate levels of tumor necrosis factor-α (88% and 93% reduction, respectively), IL-6 (98% and 94% reduction), CXCL1 (66% and 75% reduction), and CXCL2 (47% and 67% reduction). LPS-induced recruited monocyte-macrophages (CD11c^int^CD11b^+^) were reduced (68%) with rIL-10. Correspondingly, LPS-induced lung tissue CCR2^+^ inflammatory monocyte-macrophage were reduced with rIL-10. There were also reductions in LPS-induced lung neutrophils, lymphocyte subpopulations, collagen content, and vimentin expression. These findings support the importance of studying resolution processes for the development of treatment after unintended environmental/occupational biohazard exposures. Short-term, lung-delivered rIL-10 favorably hastened inflammatory recovery processes following acute, high dose inhalant LPS exposure.

## Introduction

Whereas occupational-associated lung diseases are typically caused by repeated, long-term exposure to various hazardous agents, even a robust, one-time exposure can damage the lungs resulting in long-term disease consequences. A striking example of the latter would be the lung disease that developed in first responders exposed to the dust cloud following the disastrous World-Trade Center collapse of September 11, 2001^1–3^. Whereas efforts to reduce exposures, promote respiratory protective equipment, and continue surveillance are needed, there are currently no therapies for the management of post-occupational exposure induced lung injury/inflammation, underscoring an unmet need to identify alternative approaches. Additional factors that have hampered progress consist of the complexity of the exposome including various potential occupational derivates or microorganisms^4^ as well as limited information understanding the post-exposure normative recovery and resolution of consequences in the lung. Investigations to understand the resolution processes are needed for the development of treatment options following unintended environmental and/or occupational biohazard exposures. The objective of this study was to investigate the cellular mediators of lung recovery/resolution following a standard inflammatory agent insult and investigate a potential therapeutic lung-targeted approach to reduce disease burden using an in vivo model that could be shared among investigators.

Endotoxins are characterized as lipopolysaccharides (LPS) in gram-negative bacteria with very high pro-inflammatory properties^4,5^. Human inhalant exposure to endotoxins occurs constantly, as LPS can easily bind to dust^6^. High LPS exposure induces systemic, and airway inflammatory diseases marked by influx of neutrophils, dyspnea, and chest tightness^7^. Endotoxins have been considered a main factor contributing to occupational lung diseases including textile workers, dairy workers, animal feed and grain workers, sewage treatment plant workers, and organic dust toxic syndrome^8–12^. Additional advantages of utilizing LPS as a representative inflammatory agent in experimental designs is that it is highly reproducible, commercially available, elicits a pro-inflammatory lung response in humans and rodents, and can be compared to existing published data for occupation-induced lung disease.

A potential strategy to hasten post-exposure lung recovery/resolution processes is through targeting the clinically relevant anti-inflammatory and pro-resolving interleukin (IL)-10 cytokine pathway. In a cross-sectional study of 625 veterans with farming experience, baseline IL-10 concentrations from a whole blood assay were inversely associated with ΔTNF-⍺ (− r = 0.63) and ΔIL-6 (− r = 0.37) levels with results remaining highly significant (p < 0.0001) after adjusting for potential confounders^13^. In a murine model of repetitive agriculture dust extract (LPS-enriched)-induced lung inflammation, neutrophil influx and lung pathology was elevated in IL-10 deficient mice and recombinant (r)IL-10 treatment reversed these effects^14^. Correspondingly, rIL-10 treatment reduced dust extract-induced TNF-⍺ release in cultured murine alveolar macrophages and peritoneal macrophages^14^. Prior studies also demonstrated that airway injury outcomes are increased following chronic, high concentration-endotoxin in IL-10-deficient mice^15,16^, and that airway injury was reduced when human IL-10 expression was delivered by adenoviral vector treatment to the murine lung prior to LPS exposure^16^. Therapeutic application of IL-10 (via systemic delivery) has been tested in chronic diseases including Crohn’s disease, rheumatoid arthritis, psoriasis, hepatitis C, and HIV, with partial efficacy and potential untoward reactions preventing application to clinical practice related to its long-term use^17^.

Here, we first aimed to delineate the time continuum of cell mediator events of normal recovery following acute LPS exposure, and then we hypothesized that a lung-delivered, short-term approach of IL-10 treatment could have beneficial responses following acute LPS-induced airway inflammation to hasten lung recovery.

## Methods

### Mice and exposure model

C57BL/6 mice (at 8-weeks of age) were purchased from The Jackson Laboratory (Bar Harbor, ME), randomized upon arrival, and were allowed to acclimate for one week prior to initiation of experiments. AJN, RG, and facility staff were aware of the randomization. All other authors were blinded. Animals were housed in a dedicated room away from all other animals in the facility. Male mice were utilized for all studies because we have previously demonstrated that female mice were less susceptible to inhalant endotoxin-induced airway and systemic inflammatory effects^18^. Airway inflammation was induced using intranasal or intratracheal instillation of lipopolysaccharide (LPS) of *Escherichia coli* (O55:B5) from Sigma (St. Louis, MO) whereby mice were lightly sedated under isoflurane and received treatment with either 50 µL of sterile saline or LPS (10 μg or 100 μg). Recombinant (r)IL-10 was purchased from R&D Systems (Minneapolis, MN). After determining safety dose-responses in mice, rIL-10 was intratracheally administered at 1 μg in 50 μl of sterile saline per mouse in post-LPS exposure studies. Vehicle control of 50 μl of sterile saline was also administered in side-by-side studies. Weights were recorded throughout the treatment period. At completion of experiments, mice were sedated and euthanized by isoflurane followed by exsanguination (right axillary blood collection). The study was conducted and reported in accordance with ARRIVE guidelines (https://arriveguidelines.org). Animal procedures were also approved by the University of Nebraska Medical Center (UNMC) Institutional Animal Care and Use Committee and were in accordance with the NIH guidelines for the use of rodents.

### Inflammatory marker analysis

Bronchoalveolar lavage fluid (BALF) was collected using 3 × 1 mL PBS. Total cell numbers from the combined recovered lavage were enumerated and differential cell counts determined from cytospin-prepared slides (cytopro cytocentrifuge, ELITech Group, Logan, UT) stained with DiffQuick (Siemens, Newark, DE). Lung tissue homogenates were prepared by homogenizing lung samples in 500 μl of sterile phosphate buffered saline (PBS). From cell-free supernatant of the first lavage fraction and lung tissue homogenates, tumor necrosis factor-alpha (TNF-α), IL-6, IL-10, murine neutrophil chemokines (CXCL1 and CXCL2), and the epidermal growth factor amphiregulin (AREG) were quantitated by ELISA (R&D Systems, Minneapolis, MN; minimal detectable dose (MDD) of 1.88, 1.6, 31.3, 2.0, 1.5, and 15.6 pg/ml, respectively.

### Lung cell staining and flow cytometry

Following vascular perfusion and BALF removal, harvested lungs (right lungs) were subjected to an automated dissociation procedure using a gentleMACS Dissociator instrument (Miltenyi Biotech, Auburn, CA)^19^. Lung cells from each animal were incubated with a LIVE/DEAD Fixable Blue Dead Cell Stain Kit (Invitrogen, Carlsbad, CA) and CD16/32 (Fc Block, Biolegend, San Diego, CA) to minimize non-specific antibody staining. Cells were stained with monoclonal antibody against rat anti-mouse CD45 (clone: 30-F11; BD Biosciences, Franklin Lake, NJ), CD11b (clone: M1/70; BD Biosciences), Ly6G (clone 1A8; BD Biosciences), CD11c (clone: N418; Invitrogen), CD4 (clone RM4-5, BD Biosciences), CD8 (clone 53-6.7, BD Biosciences), CD19 (clone 1D3, Invitrogen), hamster anti-mouse CD3e (clone 145-2C11, BD Biosciences), and mouse anti-mouse NK1.1 (clone PK136, BD Biosciences). Gating strategies for Ly6G^+^ neutrophils, CD3^+^CD4^+^ T cells, CD3^+^CD8^+^ T cells, CD19^+^ B cells, and NK cells (depicted in Supplemental Fig. 1) and monocyte/macrophage populations were as previously reported^18,20–24^. The percentage of all respective cell populations was determined from live CD45^+^ lung leukocytes after excluding debris and doublets. This percentage was multiplied by the respective total lung cell numbers to determine specific cell population numbers for each animal.

**Figure 1:**
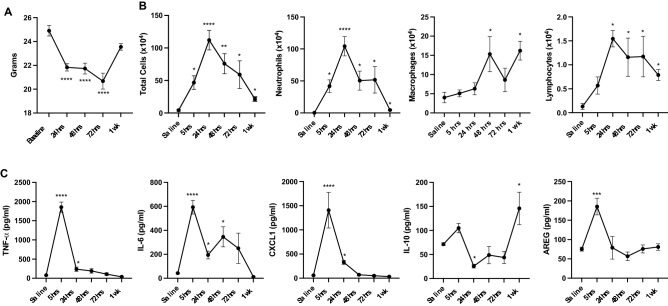
Normal homeostasis recovery trends in airway inflammatory endpoints over time following a one-time inhalation exposure to high-dose LPS. C57BL/6 mice treated once with 100 ug of LPS and allowed to recover without additional treatment up to one week. Line graphs depict mean with SEM bars over time of LPS-induced weight changes (**A**), airway cellular influx (**B**), and levels of pro-inflammatory TNF-⍺, IL-6, and CXCL1 (neutrophil chemoattractant), anti-inflammatory (IL-10), and amphiregulin (AREG) from bronchoalveolar lavage fluid (BALF). N = 24 mice (4 mice/time point). Statistical significance denoted versus saline/baseline as *p < 0.05, **p < 0.01, ***p < 0.001, ****p < 0.0001.

### Lung histopathology

Following lavage, left lungs were excised and inflated to 15 cm H_2_O pressure with 10% formalin (Fisher Scientific, Fair Lawn, NJ) for 24 h to preserve pulmonary architecture^19^. Sectioned (4–5 μm) lungs were H&E stained, and slides were reviewed at all scanning magnifications and semi-quantitatively assessed for the degree and distribution of lung inflammation by experimental pathologist (T.A.W.) blinded to the treatment conditions, utilizing a previously published scoring system^14^. This scoring system evaluates the spectrum of inflammatory changes for alveolar compartment inflammation and bronchiolar compartment inflammation. Each parameter was independently assigned a value from 0 to 5 with a higher score indicating greater inflammatory changes in the lung.

To determine CCR2^+^ inflammatory monocyte-derived macrophages, neutrophils, vimentin expression, and macrophages, lungs sections were stained with anti-CCR2 (1:100, NBP267700, Lot HM0537, Novus, Littleton, CO), anti-myeloperoxidase (MPO, 1:100, Cat#ab9535, Lot#GR331736-4; Abcam), anti-vimentin (Abcam, ab92547, Lot #GR3258719-9, 1:200), and anti-CD68 (pan-murine macrophage and scavenger receptor marker, Abcam ab31630, Lot #GR3305929-3, 1:100). Cross absorbed with donkey anti-rabbit (AlexaFluor488, A21206, Lot #2156521) or goat-anti rat AF 555 (Cat#A21434, Lot#2184321) from Thermo Fisher, Waltham, MA or goat anti-mouse IgG Alexa Fluor Plus 555 (Invitrogen A32727, Lot #UL287768) all used at 1:100 as secondary antibodies and processed as previously described^25,26^. Slices were mounted with VECTASHIELD® Antifade Mounting Medium with DAPI (4′6-diamindino-2-phenylindole; to identify nuclei)(Cat#H-1200, Lot#ZG1014, Curlingame, CA) and visualized under Zeiss fluorescent microscope. Photographs (10 per lung images per mouse) of lung parenchyma were taken from the entire section under a Zeiss fluorescent microscope (Zeiss Observer.Z1 [Zeiss, White Plains, NY]). CCR2^+^ monocyte-derived macrophages, MPO^+^ neutrophils, vimentin expression, and CD68 + macrophages were quantified by Image J FIJI plugin (version: 2.9.0/1.53t).

Lung sections were also stained with modified Masson’s Trichrome and scanned with Aperio scanner (Leica Biosystems, Deer Park, IL) by the institution’s Tissue Sciences Core Facility. VENTANA image viewer (Roche Diagnostics, Indianapolis, IN, version 3.1.4) was used to take 10 images per section at 20 × from the scanned sections. The tiff images were plugged into FIJI plugin of Image J. Collagen content in the trichrome images were quantified as described by Chen and colleagues^27^. Briefly, trichrome images were deconvoluted using the Masson’s Trichrome setting. The green component identified as the collagen fibers was used for thresholding at 170/215 to avoid cell nucleus and focus on the collagen fibers. Integrated density was measured and plotted as a measure of collagen content.

### Blood and serum studies

Whole blood collected from the axillary artery at the time of euthanasia and serum collected as previously described^19^. Blood glucose levels were determined by glucometer (True Metrix Air, Nipro Diagnostics, Fort Lauderdale, FL). Serum levels of pentraxin-2 (murine acute phase reactant protein) and IL-6 were assessed by Quantikine ELISA according to the manufacturer’s instructions (R&D, MDD of 0.159 ng/ml and 1.6 pg/ml).

### Statistical analysis

Data are presented as the mean ± standard error of mean (SEM). To detect significant changes among 3 or more groups, a one-way analysis of variance (ANOVA) was utilized and a post-hoc test (Tukey/LSD) was performed to account for multiple comparisons if the p value was < 0.05. The nonparametric Mann–Whitney test was used to detect significant changes among 2 groups. All statistical analyses were performed using GraphPad Prism (version: 9.0.0) software and statistical significance accepted at a two-sided p < 0.05.

### Ethics approval and consent to participate

Neither human participants, human data nor human tissue were used in these studies. Animal procedures were approved by the Institutional Animal Care and Use Committee and were in accordance with the NIH guidelines for the use of rodents.

## Results

### Normal homeostasis recovery trends in airway inflammatory endpoints over time following a one-time inhalation exposure to high-dose LPS

In these first set of studies, mice were intranasally treated once with 100 μg of LPS and allowed to recover without additional treatment up to 1 week. High concentration LPS induced significant weight loss at 24, 48, and 72 h (− 12% to − 17% loss, p < 0.0001) as compared to baseline with recovery of weight at one-week post-exposure (Fig. 1A). Total cellular airway influx driven by neutrophils was increased at 5 h post-exposure with peak neutrophil counts demonstrated at 24 h post-exposure, persisted to 72 h, and diminished by 1-week post-exposure (Fig. 1B). Correspondingly, airway influx of macrophages increased at 48 h and persisted at increased counts at 1-week post-exposure vs. baseline. Lymphocyte influx increased at 24 h, and lymphocyte counts remained elevated above baseline measurements at 1-week post-exposure. LPS-induced levels of pro-inflammatory TNF-⍺, IL-6, CXCL1, and amphiregulin peaked in the BALF at 5 h post-LPS exposure with a relatively rapid recovery to baseline by 24–48 h for TNF-⍺, CXCL1, and amphiregulin (Fig. 1C). IL-6 levels remained elevated until 72 h with return to baseline at 1-week post-exposure. In contrast, IL-10 levels were reduced at 48 h and increased at 1 week post exposure as compared to baseline (Fig. 1C).

### Normal homeostasis recovery trends in lung cell infiltrates over time following a one-time inhalation exposure to high-dose LPS

**Figure 2:**
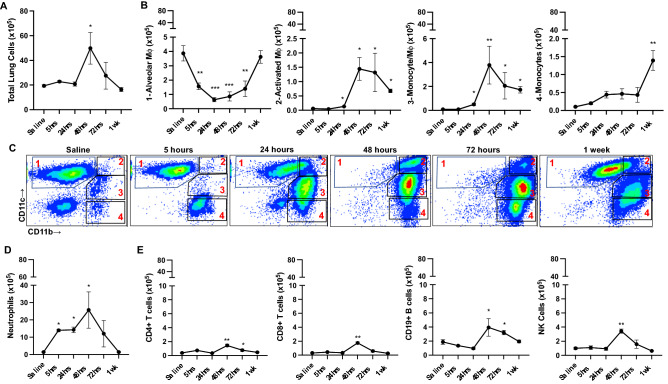
Normal homeostasis recovery trends in lung cell infiltrates over time following a one-time inhalation exposure to high-dose LPS. Lung cell populations determined by flow cytometry after removal of debris, doublets, and gating on live CD45 + cells (% population multiplied by total lung cells). Line graphs depict mean with SEM bars over time of total lung cells (**A**) and lung monocyte/macrophage subpopulations (**B**). (**C**) Gating strategy of lung monocyte/macrophage subpopulations : 1: Alveolar macrophages CD11c^+^CD11b^lo^, 2: Activated macrophages CD11c^+^CD11b^hi^, 3: Transitioning monocyte/macrophages CD11ci^nt^CD11b^hi^, 4: Monocytes CD11b^hi^ CD11c^-^ after exclusion of neutrophils. (**D**) Ly6G^+^ neutrophil, and (**E**) lymphocyte populations shown. N = 24 mice (4 mice/time point). Statistical significance denoted versus saline/baseline as *p < 0.05, **p < 0.01, ***p < 0.001.

Lung tissues were also processed for lung cell populations by flow cytometry. Total lung cell numbers increased and peaked at 48 h post-LPS exposure with return to normative numbers at 1 week (Fig. 2A). High dose LPS exposure modulated lung macrophage-monocyte populations and numbers over time (Fig. 2B,C). Namely, there were decreased numbers of alveolar macrophages (CD11c^+^CD11b^lo^) at 5, 24, 48 and 72 h with return to normative levels at 1 week. Activated/exudative macrophages (CD11c^+^C11b^hi^) increased significantly (p = 0.03) above baseline at 24 h with further rise at 48 to 72 h and remained elevated at 1-week post-exposure. At 24 h post-LPS exposure, there was an increase in the transitioning monocyte/macrophage population (CD11c^int^CD11b^hi^) that peaked at 24 h and remained increased at 72 h and 1 week. Lung Ly6G^+^ neutrophils increased at 5 h post-LPS exposure, remained elevated at 48 h, and returned to baseline values at 1 week (Fig. 2D). Numbers of lung lymphocytes including CD4 + T cells, CD8 + T cells, CD19 + B cells and NK cells demonstrated increase at 48 h-72 h with reduction to baseline values at 1 week (Fig. 2E).

Collectively, these studies describe the post-exposure time course of cellular and mediator populations in the lung up to one week following a one-time exposure to high concentration LPS and demonstrated that approximately 48 h is optimal time point for investigating lung cellular and mediator responses with a return to normal homeostasis for many endpoints except monocyte/macrophage subpopulations. Because high concentration of LPS (100 μg) induced an excessive lung inflammatory response with weight loss approaching 20% of body mass, lower concentrations of LPS (i.e., 100 ng and 10 μg) were instilled once with endpoints measured at 2 weeks post-exposure. These modified dosing studies allowed us to capture whether LPS-induced lung monocyte-macrophage populations would return to baseline/normative levels. However, even at 2 weeks post-exposure with the modified high dose LPS (10 μg) exposure, activated lung macrophages (CD11c^+^CD11b^hi^), transitioning lung monocyte/macrophages (CD11c^int^CD11b^hi^) and lung monocytes (CD11c^-^CD11b^+^) remained significantly elevated as compared to saline control (Table 1). Blood glucose levels were also significantly elevated at 2 weeks post exposure with LPS 10 μg, but not 100 ng, as compared to saline control (Table 1). There were no significant differences (p > 0.05) between LPS 10 μg and saline treatment groups in numbers of BALF cells, lung neutrophils, or lung lymphocytes at 2 weeks post-treatment (Table 1 and data not shown).

**Table 1:** Endpoints at 2 weeks following a one-time exposure to environmental inflammatory inhalant agent.

	Saline	High LPS (10 µg)
BALF Total Cells (× 10^4^)	6.3 ± 2.1	7.0 ± 2.1
BALF Neutrophils (× 10^4^)	0.024 ± 0.015	0.065 ± 0.053
BALF Mϕ (× 10^4^)	6.204 ± 2.134	6.981 ± 2.095
Lung Total Cells (× 10^5^)	4.5 ± 0.9	6.7 ± 1.4
Lung Alveolar Mϕ (× 10^5^)	1.7 ± 0.3	2.3 ± 0.3
**Lung Activated Mϕ (× 10**^**5**^**)**	0.031 ± 0.006	0.295 ± 0.059**
**Lung Monocyte-Mϕ (× 10**^**5**^**)**	0.028 ± 0.012	0.238 ± 0.0345***
**Lung Monocytes (× 10**^**5**^**)**	0.031 ± 0.010	0.149 ± 0.024**
**Blood Glucose (mg/dL)**	110 ± 22	191 ± 10*

There was no difference across treatment groups in lung neutrophils and lung lymphocyte populations. Statistical differences bolded and denoted as *p < 0.05, **p < 0.01, and ***p < 0.001. N = 3/group.

### Lung-delivered rIL-10 treatment hastens recovery of systemic and airway inflammatory events following a one-time LPS exposure

**Figure 3:**
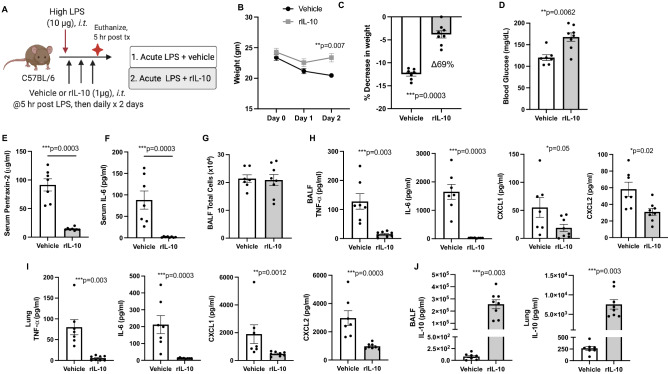
Instilled rIL10 treatment hastens recovery of systemic and airway inflammatory events following a one-time LPS exposure. (**A**) Schematic of experimental design (Created with BioRender.com). (**B**) Line graph depicts mean with SEM bars of weight changes over time. Scatter plot graphs depict mean with SEM bars between treatment groups of percent change in weight at day 3 versus baseline (**C**), blood glucose levels (**D**), serum pentraxin-2 levels (**E**), serum IL-6 levels (**F**). Total cellular influx depicted in bronchoalveolar lavage fluid (BALF) (**G**). Levels of airway inflammatory markers determined by ELISA from BALF (**H**) and lung homogenates (**I**) and levels of BALF and lung homogenate IL-10 (**J**). N = 7 (vehicle) and N = 8 (rIL-10) mice/group from 2 experimental runs. Statistical significance denoted by asterisks (*p < 0.05, **p < 0.01, ***p < 0.001, ****p < 0.0001).

In these set of studies, we sought to determine whether treatment with lung-delivered rIL-10 (1 μg) would hasten recovery from LPS-induced inflammation. Using the modified high dose LPS (10 μg) and the optimal time point of 2 days following exposure, three doses of rIL-10 or vehicle were intratracheally instilled at 5, 24, and 48 h following the one-time LPS exposure (schematic of experimental design, Fig. 3A). Treatment with rIL-10 significantly reduced the LPS-induced weight loss at day 2 (69% difference, p = 0.0003, Fig. 3B,C). Blood glucose levels were correspondingly increased in the rIL-10 treatment group as compared to vehicle control (Fig. 3D). Serum levels of the murine acute phase reactant protein, pentraxin-2, and IL-6 were both strikingly reduced with rIL-10 treatment (Fig. 3E,F, respectively). In contrast, there was no difference between groups in the numbers of total cells, neutrophils, macrophages, lymphocytes in BALF compartment (Fig. 3G and data not shown). However, airway inflammatory makers including TNF-α, IL-6, CXCL1, and CXCL2 were reduced with rIL-10 treatment, and this finding was particularly profound for TNF-α and IL-6 (Fig. 3H). These same inflammatory markers were determined in lung tissue homogenates with similar trends demonstrated (Fig. 3I). Namely, treatment with rIL-10 nearly abrogated detection of lung TNF-α and IL-6 as well as reducing CXCL1 and CXCL2 levels. Consistent with administration of IL-10, IL-10 levels were elevated in both BALF and lung homogenates (Fig. 3J).

### rIL10 treatment following a one-time high dose LPS exposure modulates specific lung monocyte/macrophage populations

**Figure 4:**
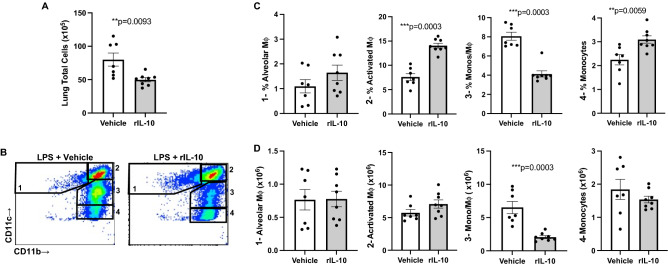
Instilled rIL10 treatment following a one-time LPS exposure modulates specific lung monocyte/macrophage populations. (**A**) Scatter plot depicts mean with SE bars of total lung cells of vehicle vs. rIL-10 treatment groups post-LPS (10 μg) exposure. (**B**) Representative image of gates for lung macrophage/monocyte subpopulations between treatment groups: 1: Alveolar macrophages CD11c^+^CD11b^lo^, 2: Activated macrophages CD11c^+^CD11b^hi^, 3: Transitioning monocyte/macrophages CD11ci^nt^CD11b^hi^, 4: Monocytes CD11b^hi^ CD11c^-^ after exclusion debris, doublets, dead cells, CD45^-^ cells, and neutrophils (Supplemental Fig. 1). Percentage of monocyte/macrophage subpopulations from live CD45^+^ lung cells depicted by scatter plot graphs (**C**). This percentage then multiplied by total lung cells enumerated to denote number of these subpopulations (**D**). N = 7 (vehicle) and N = 8 (rIL-10) mice/group from 2 experimental runs. Statistical significance denoted by asterisks (**p < 0.01, ***p < 0.001).

In these same mice, lung tissues were also processed for specific lung cell infiltrate determination by flow cytometry. First, total lung cell numbers were reduced following LPS exposure by rIL-10 treatment as compared to vehicle control (Fig. 4A). Of 4 monocyte/macrophage subpopulations examined, there was a marked reduction in the recruited, transitioning CD11c^int^CD11b^hi^ monocyte/macrophage subpopulation in mice receiving rIL-10 treatment as compared to vehicle control following LPS exposure (Fig. 4B–D). This was evident by a decrease in both the percentage (Fig. 4C) and numbers (Fig. 4D) of these CD11c^int^CD11b^hi^ cells. There was an associated increase in the percentages, but not numbers, of activated macrophages (CD11c^+^CD11b^hi^) and monocytes (CD11c^-^CD11b^hi^). There was no difference in alveolar macrophages (CD11c^+^CD11b^lo^) between groups. Correspondingly, there was also no significant difference in the expression of the murine macrophage CD68 marker (also recognized as a scavenger receptor) by immunohistochemical staining of lung sections between treatment groups (mean ± SEM, integrated density; LPS + vehicle: 5.86 × 10^4^ ± 1.81 × 10^4^, N = 7 mice/group; and LPS + rIL-10: 11.96 × 10^4^ ± 3.10 × 10^4^, N = 8 mice/group; p = 0.19).

### Effect of rIL-10 treatment following a one-time high dose LPS exposure in modulating neutrophils and lymphocyte lung populations

**Figure 5:**
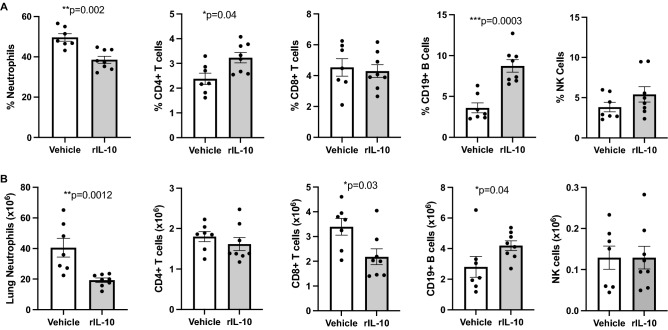
Lung-delivered rIL10 treatment following a one-time LPS exposure modulates neutrophils and lymphocyte lung populations. A, Scatter plot depicts mean with SE bars of percentage of neutrophil and lymphocyte populations of live CD45^+^ lung cells. Next, neutrophil and lymphocyte lung cell % populations multiplied by total lung cells (**B)**. Gating strategy depicted in Supplemental Fig. 1. N = 7 (vehicle) and N = 8 (rIL-10) mice/group from 2 experimental runs. Statistical significance denoted by asterisks (*p < 0.05, **p < 0.01, ***p < 0.001).

Lung-delivered rIL-10 also reduced the percentage (Fig. 5A) and number (Fig. 5B) of Ly6G^+^ lung neutrophils as compared to vehicle control following LPS exposure. For lymphocyte subpopulations, rIL-10 treatment resulted in an increase in percentage, but not number, of CD4 + T cells, decrease in number, but not percentage, of CD8 + T cells, and no difference in NK cell percentage or number (Fig. 5A,B). However, there was an increase in both percentage and number of CD19^+^ B cells following rIL-10 treatment (Fig. 5A,B).

### Lung-delivered rIL-10 treatment reduces lung histopathology, CCR2 + inflammatory monocytes/macrophages, and MPO + neutrophils

**Figure 6:**
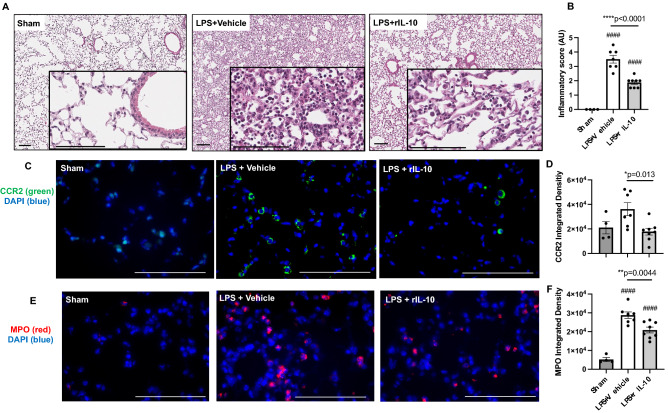
Treatment with rIL-10 reduces lung inflammation, CCR2 + inflammatory monocytes, and MPO + neutrophils following a one-time high dose LPS exposure. Representative H&E-stained lung section images from both treatment groups and sham (saline control) (**A**) with semi-quantitative lung inflammatory score (**B**) shown. Confocal images of lung tissues stained for CCR2^+^ inflammatory monocytes/macrophages (green) (**C**) and myeloperoxidase (MPO)^+^ neutrophils (red) (**E**) with nuclei staining by DAPI (blue) and integrated density of CCR2 (**D**) and MPO (**F**) quantified per each mouse. Individual values are averaged from 8 to 10 images per section per mouse. Statistical significance vs. sham (####p < 0.0001). Asterisks (*p < 0.05, **p < 0.01, ***p < 0.001). Line scale is 100 μm.

By microscopic review, there was also a reduction in acute LPS-induced bronchiolar and alveolar inflammation in mice that were treated with rIL-10 as compared to vehicle control (Fig. 6A). This histopathologic observation was confirmed by semi-quantitative assessment by pathologist blinded to treatment conditions (Fig. 6B) corresponding to the decrease in overall total lung cells with rIL-10 treatment (Fig. 4). Based upon studies demonstrating a reduction in specific LPS-induced recruited monocyte-macrophage (CD11c^int^CD11b^hi^) subpopulations with rIL-10 treatment as evident by flow cytometry studies (Fig. 4), lung sections were stained for CCR2 expression (Fig. 6C). CCR2 is a chemokine receptor primarily found on monocyte-derived macrophages and is important for monocyte-derived macrophage tracking from peripheral blood to injured tissues. By confocal microscopy, CCR2^+^ inflammatory monocyte-macrophages were reduced in rIL-10 treated mice as compared to vehicle control treated mice (Fig. 6C,D). Lung tissue MPO^+^ neutrophils were also reduced in rIL-10 treated mice as compared to vehicle control treated mice (Fig. 6E,F), consistent with the flow cytometry studies (Fig. 5).

### Lung-delivered rIL-10 treatment reduces LPS-induced lung collagen in trichrome staining and lung vimentin expression.

**Figure 7:**
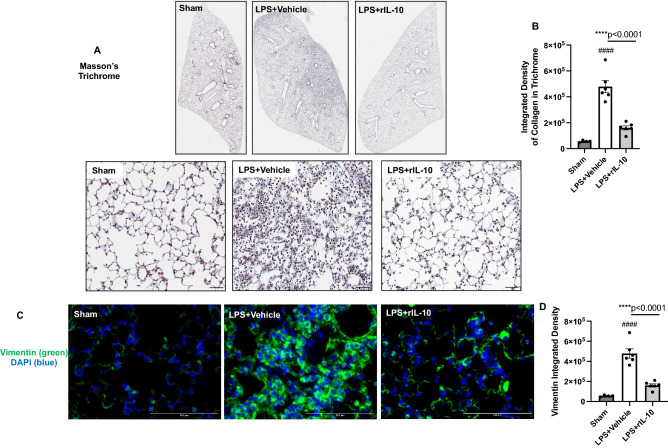
Treatment with rIL-10 reduces lung collagen and vimentin expression following a one-time high dose LPS exposure. Representative Masson’s trichrome lung section images from both treatment groups and sham (saline control) (**A**) with integrated densities from deconvoluted trichrome images as measure of collagen content (**B**) shown. Confocal images of lung tissues stained for vimentin (green) (**C**) with nuclei staining by DAPI (blue) and integrated density of vimentin (**D**) quantified per each mouse. Individual values are averaged from 8 to 10 images per section per mouse. Statistical significance vs. sham (####p < 0.0001). Asterisks (****p < 0.001). Line scale is 100 μm.

Collagen content as measured by trichrome staining was increased in LPS + vehicle treated mice as compared to the sham group, and this response was significantly reduced with lung-delivered rIL-10 therapy at 48-h post-LPS exposure (Fig. 7A,B). Furthermore, vimentin expression, an extracellular matrix protein with expression attributable to mesenchymal cells (i.e. fibroblasts, smooth muscle cells, and endothelial cells) and macrophages, was also strikingly increased post-LPS exposure versus sham mice, and this LPS-induced vimentin expression was also reduced with IL-10 therapy (Fig. 7C,D). These data support that lung-delivered IL-10 therapy post-LPS exposure reduces pro-fibrosis processes to hasten recovery.

### Lung-delivered rIL-10 treatment hastens recovery of inflammatory events without compensatory increases in pro-fibrogenic or inflammatory processes at one week following a one-time LPS exposure

In these set of studies, mice were similarly treated once with high dose LPS (10 μg) followed by three doses of rIL-10 or vehicle at 5, 24, and 48 h and then euthanized at 1-week post-LPS exposure to capture potential compensatory adverse versus resolution events with IL-10 therapy. There was no potential compensatory increase in experimental inflammatory indices, cellular recruitment, or pro-fibrogenic processes measured, and instead, several outcomes remained reduced in the IL-10 treatment group, with overall trends toward normative states in both treatment groups (Table 2). To highlight findings, there were significant reductions in the number of BALF lymphocytes and lung total cells and subpopulations of lung activated macrophages, monocyte-macrophages, monocytes, and NK cells with IL-10 therapy. LPS-induced collagen and vimentin expression (Table 2 and Supplemental Fig. 1), weight loss, and serum pentraxin levels were also reduced with IL-10 treatment. There were also no differences in lung inflammatory scores (Table 2 and Supplemental Fig. 1) or IL-10 levels from BALF and lung homogenates between groups. There was no detection of TNF-⍺, IL-6, CXCL1, CXCL2 in BALF or lung homogenates and no detection of IL-13, IL-4, or IL-12p70 in lung homogenates in LPS + vehicle or LPS + rIL-10 treatment groups (data not shown).

**Table 2:** Lung delivered rIL-10 treatment (3 doses over 2 days) hastens recovery of inflammatory events at one week following a one-time LPS exposure.

	Vehicle	rIL-10	p-value
**BALF cells** (× 10^4^)			
Total cells	31.75 ± 7.96	19.18 ± 3.29	0.42
Neutrophils	0.78 ± 0.41	0.12 ± 0.037	0.056
Macrophages	29.71 ± 8.18	18.91 ± 3.32	0.55
**Lymphocytes**	**1.25 ± 0.35**	**0.15 ± 0.10**	**0.016**
**Lung cells** (× 10^5^)			
**Total cells**	**15.14 ± 1.11**	**9.94 ± 0.64**	**0.008**
Neutrophils	0.71 ± 0.10	0.48 ± 0.09	0.095
Alveolar Mɸ, CD11c^+^CD11b^lo^	4.63 ± 0.25	4.05 ± 0.33	0.31
**Activated Mɸ, CD11c**^**+**^**CD11b**^**hi**^	**0.41 ± 0.05**	**0.12 ± 0.04**	**0.016**
**Monocyte-Mɸ, CD11c**^**int**^**CD11b**^**hi**^	**0.93 ± 0.13**	**0.16 ± 0.04**	**0.008**
**Monocytes, CD11c**^**-**^**CD11b**^**hi**^	**0.66 ± 0.12**	**0.16 ± 0.01**	**0.008**
CD4^+^ T cells	0.57 ± 0.09	0.48 ± 0.09	0.55
CD8^+^ T cells	0.33 ± 0.05	0.23 ± 0.07	0.15
CD19^+^ B cells	3.02 ± 0.25	1.88 ± 0.3	0.095
**NK cells**	**0.9 ± 0.08**	**0.48 ± 0.04**	**0.008**
Lung inflammatory score	0.4 ± 0.19	0.2 ± 0.12	0.68
Integrated density of collagen in Masson’s trichrome staining	22.04 × 10^6^ ± 1.80 × 10^6^	17.6 × 10^6^ ± 0.21 × 10^6^	0.15
**Integrated density of lung vimentin expression**	**24.81 × 10**^**4**^ ** ± 1.02 × 10**^**4**^	**10.13 × 10**^**4**^ ** ± 0.69 × 10**^**4**^**)**	**0.0079**
**IL-10** (pg/ml)			
BALF levels	124.8 ± 25.5	101.7 ± 15.1	0.84
Lung homogenate levels	1307 ± 42.6	1338 ± 80.1	0.84
**% Weight change from baseline**			
Day 1	− 9.4 ± 0.6	− 6.7 ± 0.5	0.062
**Day 2**	− **11.8 ± 0.7**	− **4.6 ± 0.7**	** < 0.0001**
**Day 3**	− **8.3 ± 1.6**	− **2.5 ± 0.6**	** < 0.0001**
**Day 4**	− **7.9 ± 1.2**	− **2.1 ± 0.9**	**0.008**
Day 6	− 0.7 ± 0.9	0.0 ± 0.8	0.99
**Serum Pentraxin (μg/ml)**	**3.26 ± 0.33**	**1.63 ± 0.12**	**0.008**

## Discussion

Occupational exposure-induced lung diseases are the primary cause of occupation-associated illness in the United States^28^. The rates of acute inhalation incidents are not well known but have been largely attributable to lack of adequate respiratory or ventilation equipment, equipment failure, leaks/spills, accidents, fires, disasters, among others with treatment consisting of supportive care approaches including oxygen, bronchodilators, and sometimes corticosteroids^29,30^. Using high concentration acute LPS inhalation animal modeling, these current time continuum studies demonstrated a rapid rise/recovery in weight loss, proinflammatory mediators, neutrophils, and lymphocytes, but also a prolonged persistence of activated macrophage and recruited/transitioning monocyte-macrophage subpopulations. Short-term, lung-delivered post-LPS exposure treatment with rIL-10 demonstrated several beneficial responses including blunting weight loss, reduction in pro-inflammatory cytokines/chemokines, reduction in lung neutrophils, reduction in lung collagen and vimentin expression, and striking reduction in recruited/transitioning monocytes-macrophages with corresponding decrease in CCR2^+^ inflammatory monocytes-macrophages. These findings support that lung-targeted IL-10 therapy could represent a potential therapeutic benefit.

Animal models are increasingly utilized to understand lung recovery/resolution processes as they can be more easily manipulated and tested. In the setting of acute LPS exposure (100 μg), others reported that lung mechanics deteriorate with the first 4 days, fully recover by day 10, and that neutrophils and classic pro-inflammatory mediators resolve quickly^31^. Those authors had also hypothesized that lymphocytes were the key cellular players in the recovery response, but their investigations failed to show a role for T cells leading them to speculate on the potential role for other cells such as macrophages^31^. Consistently, our findings demonstrated rapid recovery of TNF-⍺ (by 48 h), IL-6 (by 1 week), and murine neutrophil chemoattractant CXCL1 (by 48 h) with corresponding recovery of neutrophils, T and B lymphocytes, and NK cells by 1 week. However, the monocyte-macrophage subpopulations as defined by CD11c/CD11b expression were profoundly modulated within 24 h post-LPS exposure and these changes persisted for up to 2 weeks, the duration of the current investigations.

Lung monocytes/macrophages are considered guardians of the lung and can alter plasticity and function in response to environmental irritants to regulate lung homeostasis^32^. Although over-simplified, the classic M1-M2 macrophage dynamic encompasses opposing roles of macrophages in the lung with classically activated M1 macrophages promoting the development of acute lung injury/inflammation while activated M2 macrophages limit inflammation or can promote fibrosis^33^. The recruited and/or inflammatory lung monocyte-macrophage has emerged as driving the transition from acute inflammation to lung fibrosis^34^. Our studies strongly support the appearance, progression, and persistence of a recruited/transitional monocyte-macrophage population defined by CD11c^int^CD11b^hi^ cells first appearing at 24 h post-LPS exposure. Moreover, this subpopulation was profoundly reduced with post-exposure rIL-10 treatment. Recruited inflammatory monocyte-macrophage subpopulations have also been defined by expression of CCR2 as CCR2 is critical for mobilization of monocytes from the bone marrow to various tissues during systemic inflammation^35^. Repeated exposure to an LPS-enriched (hog barn) organic dust extract has also been previously shown to increase lung CCR2^+^ monocytes-macrophages^36^. Here, we demonstrated that rIL-10 treatment reduced tissue CCR2^+^ monocytes/macrophages following acute LPS exposure. As others have suggested more research is necessary to target pathways to minimize recruitment of these lung monocytes/macrophages (as they are long-lasting) to prevent lung fibrosis^34^, our data support that exploiting the IL-10 pathway may accomplish this goal. Furthermore, future studies to inform potential therapeutic approaches are also warranted to fully understand the phenotype (e.g. expression of various markers including F4/80, Ly6C, CD64, MHC Class II, CD80, CD86, CD14, CD206, among others), function, and potential transcriptome profile of these macrophage-monocyte subpopulations. Other proof-of principle strategies to inhibit this LPS-induced cell subpopulation include antibody blocking studies (e.g., anti-CCR2, -CD11c, -CD11b), monocyte depletion (e.g., systemic clodronate liposome reduction techniques), or potentially conditional animal knockout approaches to further understand their role.

IL-10 is a well-recognized anti-inflammatory cytokine that is naturally produced following activation of multiple pattern recognition receptor signaling pathways that exerts its functions to restrict excessive inflammatory responses and promote tissue repair^37^. In general, IL-10 exerts major suppressive effects on myeloid cells by inhibition of pro-inflammatory cytokines, directly inhibits effects on T cells, and has immune stimulatory effects on B cells^37^. Consistent with prior in vitro macrophage studies demonstrating reduction in organic dust extract-stimulated TNF-⍺ release with rIL-10^14^, lung levels of LPS-induced TNF-⍺ were essentially abrogated with rIL-10 treatment. The mechanisms of this bi-directional regulation of TNF-⍺ and IL-10 in macrophages following organic dust extract exposures was shown to be attributable to scavenger receptor A signaling pathway induction of protein kinase C zeta resulting in IL-10 release that fed-back to inhibit TNF-⍺ converting enzyme (ADAM-17) activity^14^. Others have also demonstrated that pretreatment, as opposed to post-exposure treatment, of mice with IL-10 over-expressing mesenchymal stromal/stem cells protected mice against acute lung injury to LPS and this corresponded to increased lung infiltration of IL-10 producing T cells and B cells, less weight loss, and lower TNF production^38^. Our studies also strongly support that IL-10 suppressed other pro-inflammatory mediators including IL-6 and neutrophil chemoattractants. Corresponding to the decrease in the neutrophil chemoattractants, lung neutrophils were also reduced with rIL-10 treatment. Moreover, rIL-10 therapy reduced numbers of CD8 T cell infiltrates, but increased B cell numbers, consistent with previously described IL-10 actions^37^.

Although IL-10 is well-recognized as an anti-inflammatory cytokine with ability to facilitate wound healing, it has also been suggested that the IL-10 cytokine family (e.g., IL-10, -19, -20, -22, -24, -26, -28A, -28B, and -29) also govern fibrogenesis^39^. Consistent with studies demonstrating that IL-10 inhibits the proliferation and collagen synthesis of myofibroblasts^39^, these current studies also support that lung-delivered IL-10 therapy reduced LPS-induced collagen content, and moreover, LPS-induced lung vimentin expression was also reduced. With extending studies to 1-week post-LPS exposure with and without short-term IL-10 therapy, studies did not suggest compensatory adverse or pro-fibrotic processes but supported hastening of recovery/resolution. The current studies only employed lung-directed IL-10 therapy for 2 days, and therefore, caution is necessary in future studies that might explore longer duration of treatment or use in other disease scenarios due to its potential fibrogenic properties.

Endotoxin exposure (particularly systemic) induces cachexia and weight loss and can contribute to features of the metabolic syndrome^40^. Acute LPS inhalation induced weight loss of 10–20% within the first few days with return to normal by one week. IL-10 treatment blunted LPS-induced weight loss, and this was correspondingly associated with increased blood glucose levels. Stress-related hyperglycemia is associated with increased morbidity and mortality in critical illness^41,42^, and IL-10 treatment has been shown to attenuate and prevent insulin resistance, improve insulin signaling, and beneficially affect peripheral glucose metabolism^43,44^. An explanation for the increase in blood glucose levels with rIL-10 treatment at day 2 is that it does not reflect a negative outcome, but mostly likely reflects weight differences between the 2 groups.

**Figure 8:**
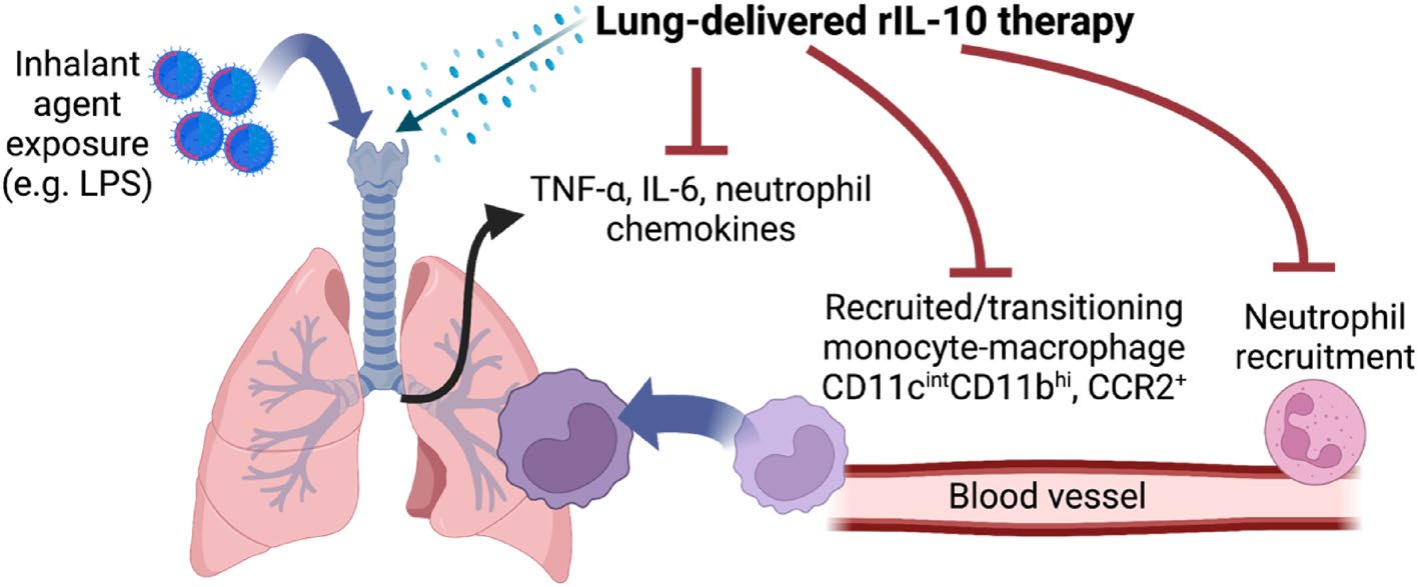
Schematic of lung-delivered rIL-10 treatment effects upon acute LPS-induced airway inflammatory consequences. Post-LPS exposure induced lung pro-inflammatory cytokine/chemokine release with corresponding recruitment of lung monocyte-macrophage subpopulation (CD11c^int^CD11b^+^) and/or CCR2 + monocytes/macrophages and neutrophils is reduced with rIL-10 therapy (Created with BioRender.com).

Although our study suggests a potential novel, lung-targeted, short-term approach to using rIL-10 therapy following airway injury/inflammation (Fig. 8), there are a few limitations that should be addressed. While we utilized LPS as a representative inflammatory agent, there are a wide number of other agents in our exposome that could be explored including industrial chemical toxins (e.g., chlorine, phosgene, hydrogen sulfide, and ammonia), heavy metals (e.g., cadmium, mercury), microbial agents (e.g., gram positive bacteria, mold spores), other real-world/complex exposures (e.g., burn pit exposures, wild-fire smoke, organic dust), and even potentially overwhelming lung infectious process as seen in viral (e.g., SARS-CoV-2) infections or bacterial infections. Whereas systemic delivery of IL-10 as treatment in various chronic diseases has shown partial efficacy, long-term use has been associated with anemia and thrombocytopenia^17^. We did not evaluate extrapulmonary organs (e.g., spleen bone marrow, liver, kidney, etc.), which should be investigated in future studies to determine potential effects. However, these potential adverse effects may be negated by short-term use applications as in settings of acute exposures (e.g., first responders, military, disasters). Next, pulmonary drug delivery options are currently limited to metered dose inhalers, nebulizers, and dry powder inhalers with recent efforts investigating novel nanoparticle formulations to maximize lung deposition and minimize pulmonary clearances of drugs^45^. Thus, future studies investigating lung-targeted IL-10 therapy should also focus on optimal lung-delivery strategies.

## Conclusion

In conclusion, the present findings strongly support the potential use of lung-delivered therapy of IL-10 in human applications, particularly in a short-term approach following untoward and extreme occupational and/or environmental inhalation exposures.

## Supplementary Information

Supplementary Information.

## Data Availability

All data generated or analyzed during this study are included in this published article [and its supplementary information files]. The datasets used and/or analyzed during the current study are available from the corresponding author on reasonable request.

## Abbreviations

LPS: Lipopolysaccharides
IL: Interleukin
R: Recombinant
BALF: Bronchoalveolar lavage fluid
PBS: Phosphate buffered saline
TNF-α: Tumor necrosis factor-alpha
AREG: Amphiregulin
MDD: Minimal detectable dose
SEM: Standard error of mean
ANOVA: A one-way analysis of variance
