# Supplementary material for: Post-endotoxin exposure-induced lung inflammation and resolution consequences beneficially impacted by lung-delivered IL-10 therapy
Source: Sci Rep. 2022 Oct 15;12:17338. doi: 10.1038/s41598-022-22346-2 (PMC9569365; doi:10.1038/s41598-022-22346-2)
Supplement: Supplementary file 1 — Supplementary Information. [file 41598_2022_22346_MOESM1_ESM.pdf]

Supplemental Figure 1

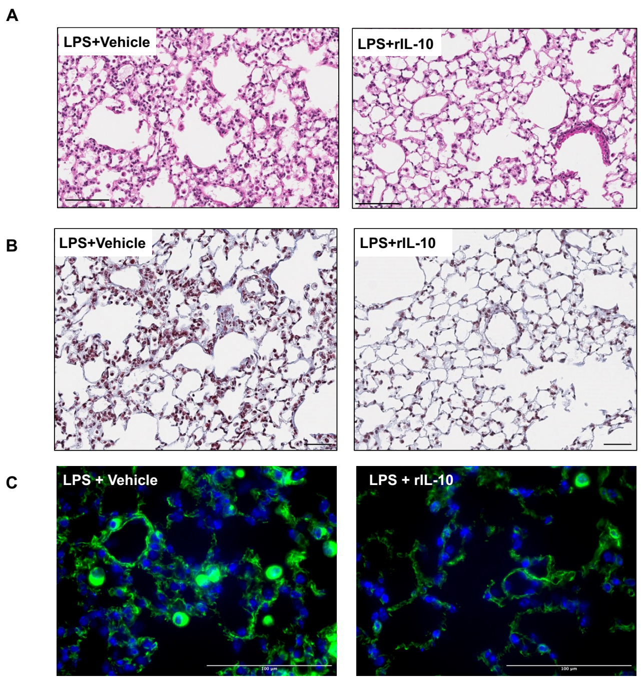

Supplemental Figure 1. Lung histopathology, collagen and vimentin expression at one week after rIL-10 treatment (3 doses over 2 days) following LPS exposure. Representative H&E **(A panel)**, Masson's trichrome for collagen content **(B panel**), and vimentin (green) with nuclei (DAPI, blue) **(C panel)** stained lung section images from both treatment groups (N=5 mice/group). Line scale is 100 µm.
